# Isolation and identification of a TetR family protein that regulates the biodesulfurization operon

**DOI:** 10.1186/s13568-019-0801-x

**Published:** 2019-05-24

**Authors:** Pooja Murarka, Tanaya Bagga, Pooja Singh, Sabita Rangra, Preeti Srivastava

**Affiliations:** Department of Biochemical Engineering & Biotechnology, Indian Institute of Technology Delhi, New Delhi, India

**Keywords:** TetR, *Gordonia* sp. IITR100, *E. coli*, *Rhodococcus erythropolis*, Biodesulfurization, Activator

## Abstract

Biodesulfurization helps in removal of sulfur from organosulfur present in petroleum fractions. All microorganisms isolated to date harbor a desulfurization operon consisting of three genes *dszA*, -*B* and -*C* which encode for monooxygenases (DszA & C) and desulfinase (DszB). Most of the studies have been carried out using dibenzothiophene as the model organosulfur compound, which is converted into 2 hydroxybiphenyl by a 4S pathway which maintains the calorific value of fuel. There are few studies reported on the regulation of this operon. However, there are no reports on the proteins which can enhance the activity of the operon. In the present study, we used in vitro and in vivo methods to identify a novel TetR family transcriptional regulator from *Gordonia* sp. IITR100 which functions as an activator of the *dsz* operon. Activation by TetR family regulator resulted in enhanced levels of desulfurization enzymes in *Gordonia* sp. IITR100. Activation was observed only when the 385 bp full length promoter was used. Upstream sequences between − 385 and − 315 were found to be responsible for activation. We provide evidence that the TetR family transcription regulator serves as an activator in other biodesulfurizing microorganisms such as *Rhodococcus erythropolis* IGTS8 and heterologous host *Escherichia coli*. This is the first report on the isolation of a possible transcriptional regulator that activates the desulfurization operon resulting in improved biodesulfurization.

**Electronic supplementary material:**

The online version of this article (10.1186/s13568-019-0801-x) contains supplementary material, which is available to authorized users.

## Introduction

Biodesulfurization is a process for removal of sulfur from organosulfur compounds present in petroleum fractions. Several biodesulfurizing microorganisms have been isolated to date from different parts of the world. All have been shown to contain desulfurization genes in the form of an operon in the order *dszA*, -*B* and -*C*. The genes *dszA* and *dszC* code for monooxygenases and *dszB* encodes a desulfinase. Thus, Dibenzothiophene (DBT) is converted into dibenzothiophene sulfone (DBT sulfone) by the enzyme DszC. DBT sulfone is converted into 2-hydroxybiphenyl sulfinic acid by the enzyme DszA, which in turn is converted into 2 hydroxybiphenyl and sulfite by DszB. There is an unlinked *dszD* gene present which encodes for a FMN oxidoreductase. DszD enzyme supplies FMNH_2_ to the flavin dependent biodesulfurization enzymes, DszA and DszC (Mohebali and Ball 2016). The *dsz* genes present in different microorganisms show 60–90% similarity (Denis-Larose et al. 1997; Oldfield et al. 1998). The genes are present in an operon under the control of a promoter. The *dsz* promoter is repressed in the presence of inorganic sulfur such as sodium sulfate and is active in presence of organosulfurs such as DBT (Li et al. 1996). According to another study by Li et al. (2008), the levels of transcription and translation of the operon genes decreased according to their position in the operon. The overlap of the genes *dszA* with *dszB* further contribute to a decrease in the expression of DszB. Li and coworkers rearranged the position of the operon genes to get an increased level of the DszB which resulted in an increase in biodesulfurization activity. While a significant number of desulfurizing microorganisms have been isolated, detailed regulatory mechanism of the *dsz* operon is unknown. Li et al. (1996) demonstrated a gel shift when *dsz* promoter was incubated with crude extract of a biodesulfurizing bacterium *R. erythropolis.* Deletion analysis of the *dsz* promoter of *R. erythropolis* showed that multiple transcription factors, activators and repressors, likely interact with the upstream region of the *dsz* operon but the identity of the proteins and the conditions under which they bind were not determined in the study (Li et al. 1996).

In the present study, we used an in vitro pull-down assay to identify the proteins that bind to the *dsz* promoter from a biodesulfurizing bacterium *Gordonia* sp. IITR100. The genes encoding the putative transcription factors were cloned and expressed in a heterologous host, *Escherichia coli*. Our findings suggest that a regulatory protein belonging to TetR family (Protein ID: WP_010840674.1) when supplied in trans (in suboptimal concentration) activates the operon and results in enhanced biodesulfurization activity in *E. coli*, *R. erythropolis* IGTS8 and *Gordonia* sp. IITR100.

## Materials and methods

### Bacterial strains and plasmids

The bacterial strains and plasmids used in the present study are presented in Table 1.

**Table 1:** Strains and plasmids used in this study

Strains or plasmid	Relevant features	Source
*E. coli* DH5α	Cloning strain	Invitrogen
BL 21(DE3)pLysS	Expression strain	Invitrogen
*Gordonia* sp. IITR100	Completely sequenced, 5.6 Mb	MCC No. 2877 (Singh and Srivastava 2013)
pET26b	Expression vector, 5.3 kb, Kan^R^, pBR322 ori, T7 promoter	Novagen, USA
pET29a	Expression vector, 5.3 kb, Kan^R^, pBR322 ori, T7 promoter	Novagen, USA
pACYC184	Promoterless plasmid, 4.2 kb, Cm^R^, Tet^R^, p15A ori	
pRSG43	Kan^R^,5.2 kb, contains pRC4 replicon	Gifted by Dr. Shavandi (Yamamoto et al. 2011)
pHYBP109	Amp^R^, contains *luxAB* genes	Gifted by Dr. Victor De Lorenzo
pTACG	Expression vector,9.2 kb, Kan^R^, *tac* promoter and *dsz* operon	This study
pNG	Kan^R^, 5.6 kb, *dsz* promoter cloned between *Nde*I and *Bgl*II restriction sites.	This study
pPM1	Kan^R^, 5.94 kb, TetR family gene cloned between *Nde*I and *Hind*III restriction sites in pET29a vector	This study
pPM2	Kan^R^,6 kb, DBRR gene cloned between *Nde*I and *Hind*III restriction sites in pET29a vector	This study
pPM3	Kan^R^, 5.92 kb, LuxR gene cloned between *Nde*I and *Hind*III restriction sites in pET29a vector	This study
pPM4	Kan^R^, 5.59 kb, XRE gene cloned between *Nde*I and *Hind*III restriction sites in pET29a vector	Murarka and Srivastava (2018)
pPM6	Kan^R^, 6.7 kb, Fis gene cloned between *Nde*I and *Hind*III restriction sites in pET29a vector	This study
pPM7	Cm^R^, 6.68 kb, TetR family gene along with T7 promoter cloned in pACYC184 vector	This study
pPM8	Kan^R^, 6.08 kb, TetR family gene cloned between *Nde*I and *Hind*III in pTAC vector	This study
pPM9	Kan^R^, 8.81 kb, shuttle vector between *E. coli* and *Gordonia*/*Rhodococcus* containing the gene for the TetR family protein	This study
pTB1	pET-26b containing3.7 kb *dsz* operon fragment cloned downstream of 385 bp *dsz* promoter, Kan^R^	This study
pTB2	pTB1 containing pSC101 ori cloned between the sites *Ssp*I and *EcoR*V, Spec^R^	This study
pTB3	pTB1 containing 385 bp *dsz* promoter fragment cloned between the sites for *Nde*I and *Fsp*I, Kan^R^	This study
pTB4	pTB1 containing 315 bp *dsz* promoter fragment cloned between the sites for *Nde*I and *Fsp*I, Kan^R^	This study
pTB5	pTB1 containing 239 bp *dsz* promoter fragment cloned between the sites for *Nde*I and *Fsp*I, Kan^R^	This study
pTB6	pTB1 containing 151 bp *dsz* promoter fragment cloned between the sites for *Nde*I and *Fsp*I, Kan^R^	This study
pTB7	pTB1 containing 63 bp *dsz* promoter fragment cloned between the sites for *Nde*I and *Fsp*I, Kan^R^	This study
pPS17	11,309 bp, Spec^R^	Srivastava et al. (2006)

### Media and growth conditions

*Gordonia* sp. IITR100 (Jaishankar et al. 2017) was cultured in minimal media containing different sulfur sources. The composition of minimal salt medium per litre was: Na_2_HPO_4_ (2.0 g), KH_2_PO_4_ (1.0 g), ammonium oxalate (4.25 g), MgCl_2_ (0.4 g) and sucrose (50 mM). Trace elements composition for 1 L was: KI (0.05 g), LiCl (0.05 g), MnCl_2_·4H_2_O (0.8 g), H_3_BO_3_ (0.5 g), ZnCl_2_ (0.1 g), CoCl_2_·6H_2_O (0.1 g), NiCl_2_·6H_2_O (0.1 g), BaCl_2_ (0.05 g), (NH_4_)_6_ Mo_7_O_24_·2H_2_O (0.05 g), SnCl_2_·2H_2_O (0.5 g), Al (OH)_3_ (0.1 g). The sulfur source used was 3 mM sodium sulfate or DBT (0.1 mM) (Konishi et al. 1997). Single colony was inoculated in 200 ml medium and incubated at 30 °C and 180 rpm for about 7 days.

For cloning and expression studies, *E. coli* was grown in LB medium at 37 °C. For the Gibbs assay, and luciferase assay, *E. coli* was grown in M9 medium containing DBT as the sulfur source. The composition of M9 medium per litre was: Na_2_HPO_4_ (6.0 g), KH_2_PO_4_ (3.0 g), ammonium chloride (1 g), NaCl (1 g), MgCl_2_ (1 mM), CaCl_2_ (1 mM) and glucose (0.4%) (Thakur et al. 2010).

The antibiotics used in the media were kanamycin (50 µg/ml), ampicillin (100 µg/ml), chloramphenicol (25 µg/ml), spectinomycin (40 µg/ml) and tetracycline (12.5 µg/ml) and were added as per the requirement. All the chemicals used were of molecular grade.

### Preparation of cell extract

Cells from both the cultures (200 ml) (*Gordonia* cells grown on minimal media containing sodium sulfate or DBT as sulfur source) were harvested at 1100×*g* for 10 min at 4 °C. The supernatant was discarded and the pellet was resuspended in 4 ml sonication buffer which consisted of 20 mM Tris–Cl, 150 mM NaCl, 0.1 mM PMSF, 1 mM DTT, 0.1 mM EDTA and 10% glycerol, pH 7.5. Sonication was performed on Q125 sonicator (Qsonica sonicators, USA) by giving 20 s pulse on and 30 s pulse off for 10 min at an amplitude of 50%. After sonication, the cellular debris was removed by centrifugation at 12,900×*g* for 20 min at 4 °C. The supernatant was collected in a clean microcentrifuge tube and used for further experiments.

### Pull down assay

To isolate the protein(s) binding to the DNA of our interest, pull down assay was performed. The *dsz* promoter region was amplified by PCR using primer PS18 and 5′ biotin labeled PS19 primer (Table 2). The biotinylated promoter was attached to streptavidin resins by incubating both for 10 min in binding buffer (12% glycerol, 12 mM HEPES, 4 mM Tris–Cl, 60 mM KCl, 1 mM EDTA and 1 mM DTT, pH 7.9). To this complex, crude protein extract of the bacteria was added and was incubated for 30 min with continuous rotation in a cold room. The unbound proteins were removed by washing with binding buffer and the bound proteins were eluted with elution buffer (12% glycerol, 20 mM Tris–Cl, 1 M KCl, 5 mM MgCl_2_, 1 mM EDTA, 1 mM DTT, 20 μg/ml BSA, pH 5.8). The pull-down assay was also performed using streptavidin coated dynabeads. In this assay, the biotinylated promoter was attached to streptavidin dynabeads. To this complex, the crude protein extract was passed and the elution fractions were collected by placing the microcentrifuge tube containing the reaction mixture in a magnetic rack.

**Table 2:** List of primers used in the study (restriction sites italicized)

Primer Name	Resulted fragment	Sequence (5′→3′)
GAA-F	*DszA*	GGAATTCC*ATATG*GCTCAACGGCAACAACTGCATCTGGCG GTTTC
GAA-R	*DszA*	CCG*CTCGAG*GTGTGTCGAGGTGTGTCGAGGATGCCGGTA TCAAGTTCTGAACCGG
PS-18	*Pdsz* F	GAGC*AGATCT*GGCCATGATCGACCGCCTCGTCCATCACGC
PS-19	*Pdsz* R	CAGT*CATATG*CGCGTATGTGTCCTCTAACCGTAAATAGCG
TB1	385 bp P*dsz*	GATC*GATCT*CGCGGCCATGATCGACCGCCTCG
TB2	315 bp P*dsz*	GATC*GAGTCT*ACCGAGACCTGGGCCGCGTCC
TB3	239 bp P*dsz*	GATC*GAGTCT*GTCAACTTTCAACCGCCGAAAAGGGGTGAGATTTCAGCC
TB4	151 bp P*dsz*	GATC*GAGTCT*CCACCGTTAATCTGACAGTCCCGCCCGAACTGCTG
TB5	63 bp P*dsz*	GATC*GAGTCT*GGGGTGACACTTCTTGGCGACACGAAGCACTCC
PS18 EcoRV	385 bp P*dsz*	*GAGCGATATCGGCCATGATCGACCGCCTCGTCCATCACGC*
TetR-F	681 bp TetR F	CTAG*CATATG*TTGTCACCACGAGGCCAGAC
TetR-R	681 bp TetR R	CTAG*AAGCTT*GCGCGGCAGGATGCCGTCGAG
DBRR-F	687 bp DBRR F	CTAG*CATATG*ATGAGCACGGACAAGACACGCGTCC
DBRR-R	687 bp DBRR R	CTAG*AAGCTT*TCACGGCGCCCCGCGAACGAACCGG
LuxR-F	684 bp LuxR F	CTAG*CATATG*GTGCCGATCACCGTAGTCATCGCAG
LuxR-R	684 bp LuxR R	CTAG*AAGCTT*TCACTTGATGCCGTTCTCGTAGGCG
Fis-F	1467 bp Fis F	CTAG*CATATG*ATGGCACGACCCCGGAAGCCTGAAGACCCA
Fis-R	1467 bp Fis R	CTAG*AAGCTT*ATCCAGACGCGTGGCGATGTATCGGGAGTA
qPCR-dszB	116 bp *dszB* F	GCGTCTACTCGGCATCAC
qPCR-dszB	116 bp *dszB* R	CCGAAGCCGACACTCCTATT
qPCR-dszC	94 bp *dszC* F	GCCTTCATTGTCGCCTTCAT
qPCR-dszC	94 bp *dszC* R	GCGCGATCCCTAAATAGACG

The eluates from the pull down assays were run on 12% SDS-PAGE gel with 5% stacking gel (Laemmli 1970). The gel was stained with coomassie brilliant blue and the different bands observed in the eluted fractions were cut and in-gel digested with trypsin following the protocol described by Bruker Daltonics, Bremen, Germany adapted from Shevchenko et al. (1996). The digested peptides were dried in a speed vac and the samples were analyzed by MS/MS on ABI Sciex 5800 TOF/TOF system, USA.

### Cloning and expression of proteins identified in pull down assay in pET29a vector

All recombinant DNA methods were performed following the protocols from Sambrook and Russel (2001). Plasmid pET29a was used to clone gene encoding for different proteins under the control of T7 promoter. The gene for the protein was PCR amplified from genomic DNA of *Gordonia* sp. IITR100 with the primers containing restriction sites for *Nde*I and *Hind*III. Genomic DNA from *Gordonia* sp. IITR100 was prepared following the protocol by Singh et al. (2016). The amplified PCR product and vector was digested with the same enzymes and the clone was confirmed by insert release (plasmids pPM1–pPM6). The recombinant plasmid was transformed to different expression strains to overexpress the protein of interest. The constructed plasmid was also transformed along with plasmids pTB2 and pHYBP109 in BL 21 (DE3) plysS cells to develop a three-plasmid system which was used for luciferase assay.

### Cloning of the TetR family protein into pTAC (pPOS29) and pACYC184 vector

The gene for the TetR family of transcriptional regulator was also cloned under *tac* promoter between the restriction sites *Nde*I and *Hind*III in plasmid pTACG (pPOS29). The constructed plasmid was named pPM8. Clone was confirmed by insert release and overexpression was checked in BL21 (DE3) pLysS after induction with 1 mM IPTG. The plasmid was used to make a shuttle vector for the expression of gene encoding for the TetR family protein.

The TetR family protein was also cloned in pACYC184 vector along with T7 promoter from pET29a. For this purpose, pACYC184 plasmid was digested with restriction enzymes *Nru*I and *EcoR*V. The large 3458 bp fragment was gel eluted. Plasmid pPM1 was digested with enzymes *Fsp*I and *Ssp*I. Fragment of size 3164 bp was gel eluted. Both the eluted fragments were ligated, transformed in DH5α cells and colonies screened by colony PCR. Recombinant plasmid of 6.68 kb was confirmed by release of insert. The plasmid constructed was named pPM7. The constructed plasmid was used along with plasmids pTB2 and pHYBP109 to transform BL 21 (DE3) plysS cells. The transformants obtained were used for luciferase assay.

### Construction of shuttle vector

A shuttle vector for expression of the TetR family gene in *E. coli* and Gram-positive bacteria (*Gordonia* and *Rhodococcus*) was constructed. For this, the pRC4 replicon was obtained by digesting pRSG43 plasmid (Yamamoto et al. 2011) with restriction enzymes *Pst*I and *Fsp*I. The large fragment of size ~ 5 kb was gel eluted. The gene encoding for the TetR family protein along with *tac* promoter was obtained by digesting plasmid pPM8 with restriction enzymes *EcoR*V and *Fsp*I. The large fragment of size ~ 4462 bp was gel eluted. The eluted fragments were ligated to generate a construct of size ~ 9 kb (pPM9) which can replicate in both *E. coli* and members of Actinobacteria (*Gordonia* and *R. erythropolis* IGTS8). The plasmid was used for the expression of the TetR family protein in *Gordonia* and *R. erythropolis* IGTS8.

Electrocompetent cells of *Gordonia* sp. IITR100 were prepared by following the protocol by Singh and Srivastava (2013). To prepare the competent cells of *R. erythropolis* IGTS8, single colony of the bacterium was inoculated in 100 ml LB medium and allowed to grow at 30 °C until its OD_600_ reached 0.5. Cells were harvested by centrifugation at 1100×*g* for 10 min followed by washing with ice cold 10% glycerol twice. Finally, the cells were resuspended in 1 ml 10% glycerol and the aliquots of 100 µl were stored in − 80 °C until further use.

### Construction of plasmids pTB1 and pTB2

Plasmid pNG contains the native *dsz* promoter cloned between recognition sites for *Nde*I and *Bgl*II in pET-26b. This plasmid was digested with *Nde*I and *Hind*III. The fragment of about 5.5 kb was gel purified. Plasmid pTACG contains *dsz* operon cloned between recognition sites for *Nde*I and *Hind*III downstream to the *tac* promoter. Plasmid pTACG was digested with *Nde*I and *Hind*III and the fragment of ~ 3.7 kb was gel eluted. The purified vector and insert were ligated and transformed into *E. coli* DH5α competent cells. Colony PCR was performed using primers GAA-F and GAA-R (gene specific primers of *dszA* gene). Clones were further confirmed by performing colony PCR using PS-18 and PS-19 primers (gene specific primers of native *dsz* promoter). Positive clones were further confirmed by restriction mapping. The plasmid constructed was named pTB1.

The pBR322 ori in pTB1 was replaced with pSC101 ori to get the plasmid pTB2. For this purpose, plasmid pTB1 containing the pBR322 ori was isolated and digested with *Ssp*I and *EcoR*V. The fragment of approx. 6.4 kb was gel purified. Plasmid pPS17 containing the pSC101 ori was isolated and digested with *Ssp*I. The fragment of about 5.09 kb was gel eluted. The gel purified vector and insert were ligated and transformed into *E. coli* DH5α competent cells. Clone was confirmed by colony PCR and restriction digestion. The constructed plasmid was used as a source of the *dsz* operon for desulfurization studies in *E. coli*.

### Construction of deletion mutants

For making the 5′ deletions in the full-length promoter, PCR was performed using pTB1 as template and forward primer (TB1–TB5) and PS-19 as the reverse primer. The amplicons obtained were of sizes 385 bp, 315 bp, 239 bp, 151 bp and 63 bp. The resection of the promoter (done by PCR) was in the distal to proximal direction. The amplicons were digested with *Nde*I and purified using PCR purification kit (Qiagen, Germany).

Plasmid pTB1 was digested with *Nde*I and *Fsp*I and a fragment of ~ 7061 bp was gel eluted. Each of the digested PCR products was cloned into digested plasmid pTB1, generating constructs pTB3–pTB8.

### Gibbs assay

The desulfurization activity was monitored using the Gibbs assay. In Gibbs assay, the amount of 2HBP produced is determined which is the end product of the desulfurization by 4S pathway. *E. coli* cells harboring pTB2 plasmid and pPM1 plasmid were grown in M9 medium containing DBT as the sulfur source until OD_600_ ~ 0.5. The culture was induced with IPTG and samples were collected at different time intervals. The pH of 1 ml of culture was adjusted to 8 with 10% (w/v) sodium carbonate and 10 µl of Gibbs reagent (1% 2,6 Dichloroquinone-4-chloroimide in ethanol) was added. The mixture was incubated at 30 °C for 30 min in dark. The cell suspension was centrifuged at 8960×*g* for 2 min to remove the cells and the absorbance of the supernatant was determined at OD_610_. Similarly, a culture with uninduced TetR family protein was prepared. As a control experiment *E. coli* cells harboring pTB2 plasmid was used (Konishi et al. 1997).

### Luciferase assay

Luciferase assay was performed using luciferase assay kit from Agilent Tech, USA. Briefly, *E. coli* cells harboring plasmids pHYBP109, pTB2 and pPM7 were grown in M9 medium containing DBT as the sulfur source until OD_600_ ~ 1. In another set of experiment, the cells containing the above three plasmids were grown in the same medium and induced with 0.2 mM IPTG when the OD_600_ ~ 0.5. The cells were harvested by centrifugation at 8960×*g* for 2 min and were then resuspended in resuspension buffer (50 mM glucose, 25 mM Tris HCl (pH 8) and 10 mM EDTA (pH 8). To this 3 µl lysozyme (from 45 mg/ml stock) and 1 µl RNase (from 10 mg/ml stock) was added and incubated at 37 °C for 1 h. PMSF (final concentration 1 mM) was added to the above culture and incubated in ice for 30 min. The cell lysate was centrifuged at 8960×*g* for 10 min and the supernatant was collected in a fresh tube. Supernatant (70 µl) was mixed with 30 µl of luciferase substrate assay buffer mix and immediately put in luminometer. The light produced from the reaction ~ 2 s after adding the supernatant was measured for 10 s and the final value in RLU/s was recorded. The delay time for all the samples was kept same.

Similar protocol was followed for luciferase assay with other cloned proteins.

### RT-PCR

To determine the effect of TetR family protein on the *dsz* operon at transcript level, RT-PCR was performed. *E. coli* cells harboring plasmids pTB2 and pPM1 were grown in M9 medium containing DBT as the sulfur source until OD_600_ ~ 0.5. The culture was induced with IPTG (final concentration 0.2 mM) and was allowed to grow until its OD_600_ ~ 1. Total RNA was isolated from these cells using Gsure bacterial RNA isolation kit (GCC Biotech, India) according to manufacturer’s protocol. About 1 µg of the isolated RNA was converted to cDNA using cDNA synthesis kit (Thermo Fischer scientific, USA). RT-PCR reaction was set up using the above prepared cDNA as a template and primers for intragenic region of *dszB* and *dszC* (Table 2). Fold change in the expression of *dsz* genes was calculated by double delta C_T_ method. As a control experiment *E. coli* cells harboring pTB2 plasmid was used.

Similar protocol was followed to isolate RNA from *Gordonia* grown in media containing DBT and sodium sulfate as the sole sulfur source respectively. RT-PCR reaction was set up using the above prepared cDNA as a template and primers for intragenic region of the TetR family protein (Table 2). Fold change in the expression of the TetR family gene was calculated by double delta C_T_ method.

## Results

### Isolation and cloning of P*dsz* binding proteins

**Fig. 1:**
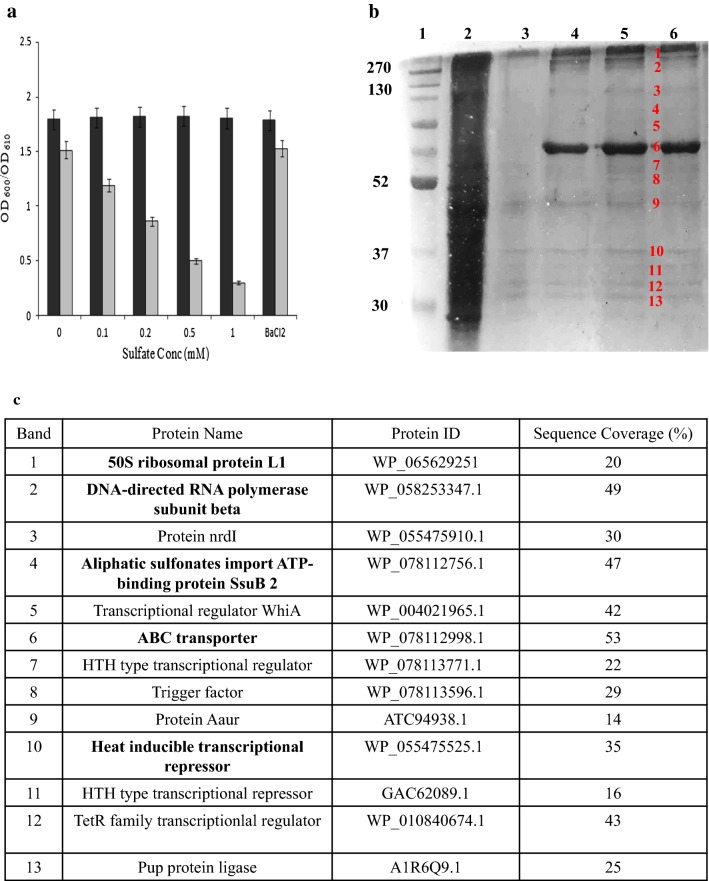
**a** Graph showing the effect of inorganic sulfate on desulfurization activity of bacterium. Black bars represent growth and gray bars represent activity of the bacterium, *Gordonia* sp. IITR100. **b** A 12% SDS-PAGE gel showing the different fractions in pull down assay with sodium sulfate extract. Lane 1: broad range protein marker lane 2: crude extract, lane 3: flow through from column, lane 4–6: elution fractions. **c** A table showing the major protein identified corresponding to each band. The proteins marked in bold were common to proteins obtained in pull down assay using kanamycin promoter (control)

To isolate the protein(s) that bind to *dsz* promoter (385 bp) from *Gordonia* sp. IITR100, pull down assay was performed using crude cell extracts. Since the promoter is repressed in the presence of inorganic sulfur and is turned on in the presence of organosulfur (Fig. 1a), two different cell extracts of the bacterium *Gordonia* sp. IITR100 were used; one was prepared by growing the cells in an organosulfur compound, (DBT) and the other in sodium sulfate. About 13 bands were detected in the elution lane on an SDS PAGE when extract from sodium sulfate grown cells was used (Fig. 1b). DNA binding proteins usually contain helix turn helix (HTH) motif. Thus, a number of transcription regulators containing HTH motif were identified in these bands. They belonged to WhiA and TetR family of transcription regulators. About 10 bands were detected when extract from DBT grown cells was used. A DNA binding response regulator and an uncharacterized transcription regulator were detected in eluted samples from DBT grown cell extract.

To eliminate non-specific DNA binding proteins, a control experiment was setup with kanamycin promoter. The proteins identified are listed in bold in Fig. 1c.

A common list of possible regulatory proteins was prepared which was largely based on the following considerations: (1) DNA binding proteins generally contain a helix turn helix motif; (2) The proteins were classified based on the type of transcription factor family and associated function, thus protein families known for regulating catabolic genes were selected e.g. TetR family over other protein families; (3) The proteins which were detected in both in vitro and in vivo assays (Murarka and Srivastava 2018) were also selected (e.g. TetR family protein was detected in in vivo assay also); (4) The genes encoding for regulatory proteins as observed in literature are usually present near the operon, since the genome sequence of *Gordonia* sp. IITR100 was determined recently, a transcription regulator, belonging to the Fis family, which were detected approximately 5–6 kb upstream of the operon was also selected.

Considering all the above factors, five genes encoding for the various DNA binding proteins were selected for cloning. These proteins belonged to different families of transcriptional regulators which are TetR family (Protein ID: WP_010840674.1), LuxR (protein ID: WP_078114038.1, DNA binding response regulator (protein ID: WP_078111994.1), XRE family protein (protein ID: WP_078114162.1) and Fis family of protein (protein ID: WP_078112001.1). The genes were cloned in an *E. coli* plasmid pET29a. Maximum protein expression was found in BL21(DE3) pLysS after 5 h of induction with 1 mM IPTG. A representative gel showing expression of the TetR family protein is shown in Additional file 1: Figure S1A. Also, it was observed that considerable amount of protein was present in soluble fraction after the optimum induction time (Additional file 1: Figure S1B). For further confirmation, the induced band was cut and the protein was identified by Peptide mass finger printing (PMF). PMF for TetR family transcriptional regulator is shown in Additional file 1: Figure S2.

### Biodesulfurization activity in the presence of transcription regulators

In order to determine the effect of expression of these proteins on the *dsz* promoter which directs the expression of biodesulfurization genes, a heterologous host *E. coli* was used and a two-plasmid system was constructed consisting of pTB2 carrying the native *dsz* operon and the other plasmid containing the gene encoding for a possible transcription factor chosen above. Formation of 2 hydroxybiphenyl (Additional file 1: Figure S3A) was detected by Gibbs assay at various time points.

The results revealed that biodesulfurization activities were enhanced only in case of plasmid pPM1 which contained a gene encoding for TetR family transcription regulator containing helix turn helix motif. It is noteworthy to mention that the biodesulfurization activities were higher even in uninduced cells expressing the TetR family transcription regulator suggesting that the protein is required at a very low concentration to activate expression of the *dsz* operon (Additional file 1: Figure S3B).

**Fig. 2:**
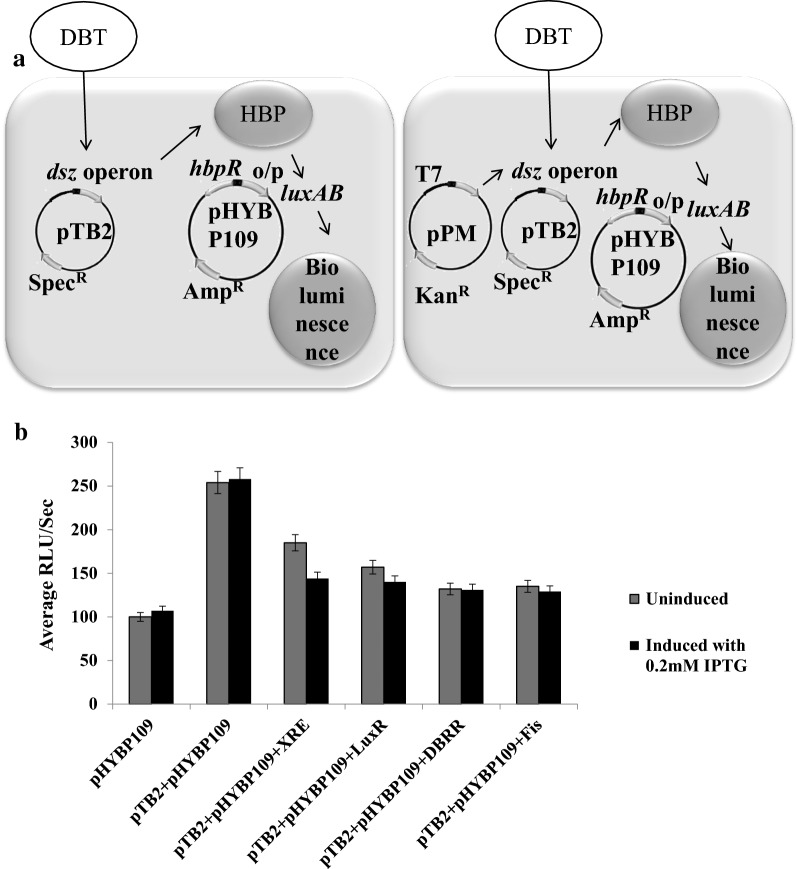
Biodesulfurization activity in *E. coli* BL21(DE3) pLysS when cloned proteins is supplied in trans via plasmid as demonstrated by luciferase assay. **a** Schematic diagram of the assay; **b** Graph showing that there is no increase in activity of the *dsz* operon, rather they are slightly reduced. The grey bar indicates the uninduced sample and the black bar indicates the luminometer reading when the cells are induced with 0.2 mM IPTG. The experiment was performed at OD_600_ ~ 1 in triplicates

In order to determine the intracellular 2-hydroxybiphenyl (2-HBP) which should be directly proportional to the levels of the desulfurization enzymes, a third plasmid pHYBP109 was introduced. In the plasmid pHYBP109, the *luxAB* reporter gene coding for bioluminescent bacterial luciferase, is placed under the control of *hbpc* promoter (P*hbpC*). PhbpC is activated in response to 2-HBP, thus the *lux* transcript fused to P*hbpC* is upregulated in parallel to the concentration of 2-HBP. In the three-plasmid-containing system, 2-HBP is produced by the recombinant *E. coli* host carrying the plasmid pTB2 containing the *dsz* operon. Here, the three-plasmid system was used to determine increase in luciferase activity which in turn is due to increase in 2-HBP levels as a result of the activation of *dsz* operon by TetR family transcription regulator (Fig. 2a) (Jaspers et al. 2000). Luciferase assay was performed with cloned proteins such as Fis, DNA binding response regulator and LuxR. Another transcription regulator belonging to XRE family was also used (Murarka and Srivastava 2018). XRE family transcription regulator was detected by an in vivo based assay and has been shown to bind to the *dsz* promoter (Murarka and Srivastava 2018) (Fig. 2b). None of these proteins resulted in increased biodesulfurization activity. Therefore, further experiments were performed with the TetR family protein. As another control, plasmid pHYBP109 was used along with plasmid expressing TetR family protein. These cells were not able to grow in DBT as sole sulfur source because of the absence of pTB2 which contains the *dsz* operon.

**Fig. 3:**
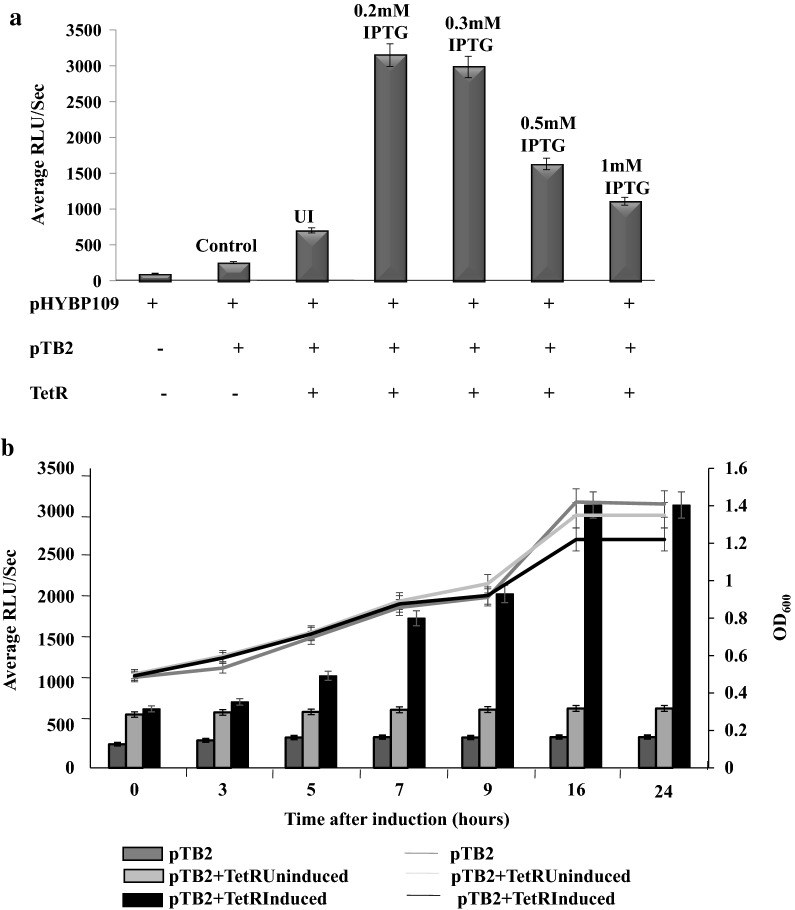
Biodesulfurization activity in *E. coli* BL21(DE3) pLysS when TetR family protein is supplied in trans via plasmid demonstrated by luciferase assay. **a** Graph showing the effect of different inducer concentrations (which corresponds to different TetR protein levels) on the luciferase activity. UI corresponds to uninduced samples. **b** Graph showing that TetR family protein expression (at 0.2 mM inducer concentration) does not affect growth and results in enhanced luciferase activity. The bar indicates the values obtained in luminometer and the lines indicate the growth (OD_600_). The experiment was performed in triplicates

With only pTB2 and plasmid pHYBP109, the luciferase activities in recombinant *E. coli* were about 200 RLU/s. Luciferase activity was measured when the TetR family protein was induced with various IPTG concentrations (0.2 mM, 0.3 mM, 0.5 mM and 1 mM IPTG). Expression of the TetR family protein was determined at these various inducer concentrations (Additional file 1: Figure S4). The maximum activity was observed when TetR family protein was induced with 0.2 mM IPTG (Fig. 3a). At this inducer concentration, the luciferase activities increased up to 3000 RLU/s (15-fold increase). Further, the luciferase activities were compared in uninduced cells and induced with 0.2 mM IPTG and the effect on growth was monitored (Fig. 3b). These results clearly indicate that the TetR family protein is involved in transcriptional activation of the *dsz* operon. In the presence of the TetR family protein (induced to IPTG concentration 0.2 mM), the growth of the cell is not affected (Fig. 3b). Similar to the results of Gibbs assay, it is noteworthy to mention that the biodesulfurization activities were higher (up to 700 RLU/s) even in uninduced cells expressing the TetR family transcription regulator.

### Expression of *dsz* genes in *E. coli* in the presence of TetR family protein

**Fig. 4:**
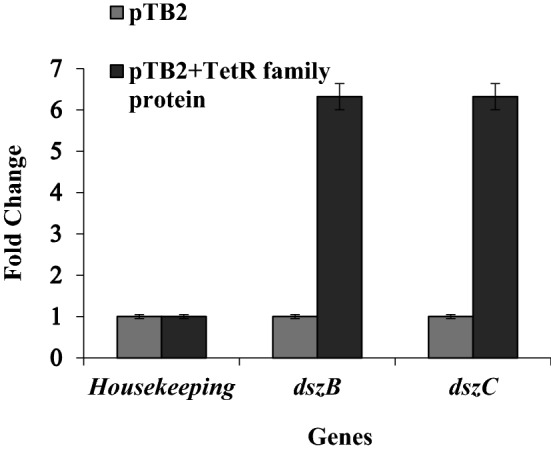
Graph representing an increase in expression of *dsz* genes (*dszB* and *dszC*) in presence of TetR family protein through RT-PCR. The experiment was performed in triplicates and repeated twice

To further validate the effect of TetR family protein on the *dsz* operon, changes at transcript level of the *dsz* operon genes was studied by RT-PCR. RNA was isolated from *E. coli* cells containing pTB2 plasmid and pPM1, cDNA was prepared and C_T_ values were determined by RT-PCR. On calculation of fold change by double delta C_T_ method, a sixfold increase in the level of *dszB* and *dszC* genes was found in presence of TetR family protein. As a validation of RT-PCR data, gyrase gene was used. C_T_ value of gyrase gene was found to be constant both in the presence and absence of TetR family protein (Fig. 4).

### Biodesulfurization activities in recombinant *Gordonia* and *Rhodococcus erythropolis* IGTS8

**Fig. 5:**
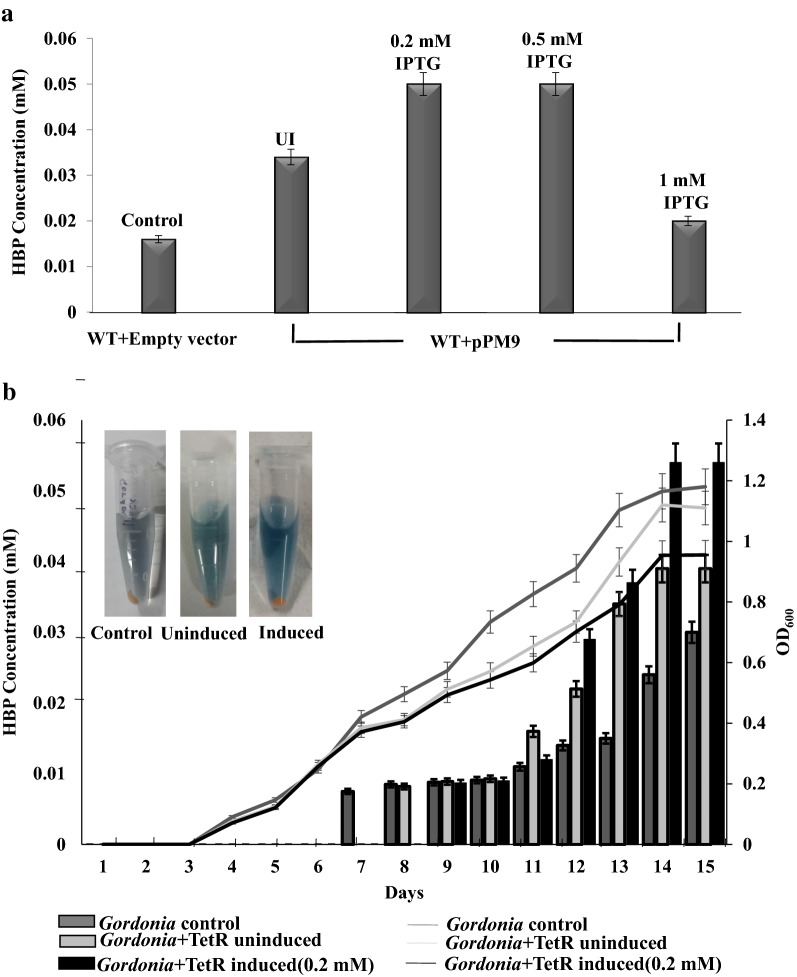
Biodesulfurization activities in *Gordonia* sp. IITR100 when grown in DBT containing medium are enhanced when the TetR family protein is supplied in trans via plasmid. **a** Graph showing the effect of different inducer concentrations on the activity of *dsz* operon in *Gordonia*. UI corresponds to uninduced samples. **b** Graph showing an increase in activity of the *dsz* operon when the TetR family protein is induced with 0.2 mM IPTG. The bars represent 2 HBP concentration and the lines represent growth

*Gordonia* sp. IITR100 harboring the shuttle plasmid pPM9 which expresses the TetR family regulator showed enhanced desulfurization activities. The activity was maximum when TetR family transcription regulator was induced with 0.2 mM IPTG. About 3.6-fold increase in desulfurization activity was observed when cells of same OD_600_ ~ 1 were compared (Fig. 5a, b). Similar to the results in *E. coli*, an increase in biodesulfurization activity was observed in uninduced condition when the protein is supplied through plasmid containing low copy number pRC4 replicon (Hashimoto et al. 1992). Increased formation of blue colored metabolite was clearly visible in case of uninduced cells and induced with 0.2 mM IPTG (Fig. 5). To determine the effect of the TetR family protein expression on growth, wild type *Gordonia* harboring empty vector and *Gordonia* harboring vector expressing the TetR family regulator were used. No effect on growth was observed when uninduced cells or induced with 0.2 mM IPTG were used (Fig. 5).

**Fig. 6:**
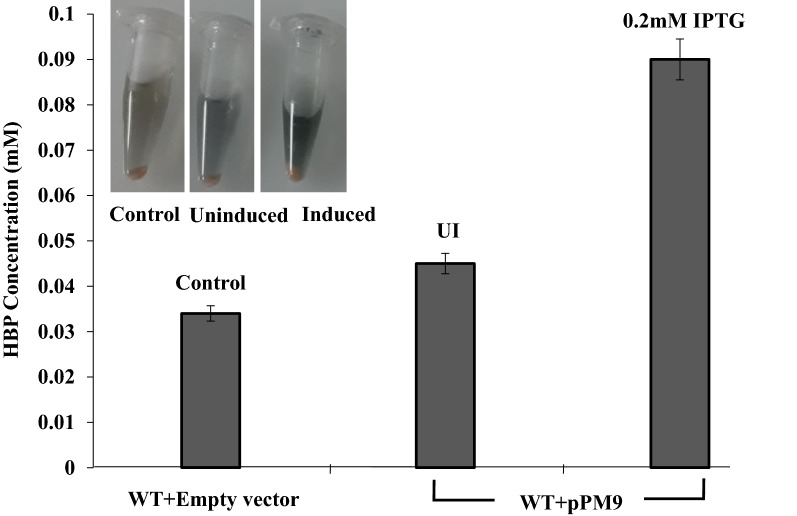
Graph showing an increase in activity of the *dsz* operon in *Rhodococcus erythropolis* IGTS8 when the TetR family protein is induced with 0.2 mM IPTG

To determine whether the TetR family transcription regulator serves as an activator of desulfurization operon in other biodesulfurizing bacteria, *Rhodococcus erythropolis* IGTS8 was used. *R. erythropolis* containing plasmid pPM9 showed enhanced biodesulfurization as compared to wild type cells (Fig. 6). About 2.6-fold increase in biodesulfurization activity was observed in cells containing TetR family protein expression plasmid induced with 0.2 mM IPTG suggesting that the mechanism of activation is conserved across various genera.

### Enhanced levels of Dsz enzymes in recombinant *Gordonia*

**Fig. 7:**
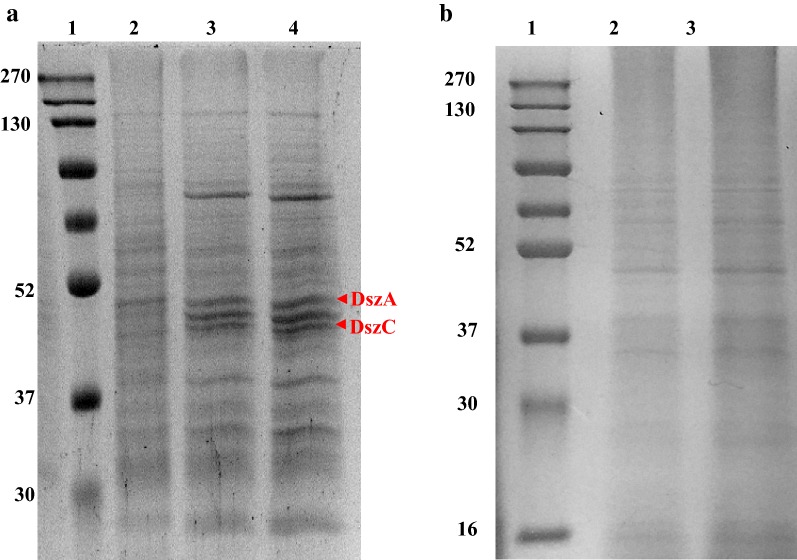
**a** A 12% SDS-PAGE gel showing expression of Dsz proteins (DszA ~ 52 kDa, DszC ~ 45 kDa) in presence of the TetR family protein when the cells are grown in DBT containing medium. Lane1: marker, lane2: Wild type *Gordonia* (sonicated supernatant), lane3: *Gordonia* transformed with the TetR family protein uninduced (sonicated supernatant), lane4: *Gordonia* transformed with the TetR family protein induced (sonicated supernatant). The proteins were identified by MALDI-ToF. **b** A 12% SDS-PAGE gel showing the expression of Dsz proteins when cells are grown in DBT containing medium. Lane1: marker, lane2: Wild type *Gordonia* (sonicated supernatant) when induced with IPTG at Day 0, lane 3: Wild type *Gordonia* (sonicated supernatant) when induced at OD_600_ is 0.5. Induction of the *dsz* enzymes are not observed in absence of the TetR family protein

In order to confirm that the enhanced desulfurization activities are attributed to the enhanced Dsz protein levels, *Gordonia* cell extract from wild type cells, uninduced and induced with 0.2 mM IPTG was prepared and run on an SDS PAGE. It was found that the Dsz protein levels increased in cells expressing TetR family transcriptional regulator (Fig. 7a). Two of the bands were identified as DszA and DszC proteins by Peptide mass fingerprinting. Two more bands corresponding to molecular weights 41 kDa and 48 kDa were also induced. They were identified as glutamate dehydrogenase and elongation factor respectively. Two sets of control were used for this experiment. In the first, IPTG was added to the wild type *Gordonia* cells when grown in media containing DBT at day 0 and in another set, IPTG was added to the wild type *Gordonia* cells when grown in media containing DBT when OD_600_ ~ 0.5. No expression of the Dsz enzymes were found in these conditions (Fig. 7b).

The preceding work was carried out using full-length 385 bp *dsz* promoter. In order to determine a minimum region required for activation by the TetR family transcription factor, deletion analysis of the *dsz* promoter region was conducted.

### Identification of the minimal promoter and determination of region required for activation

For determination of minimal promoter, the same two-plasmid system was used as described above which can detect the amount of DBT being converted to 2-HBP in vivo. This was based on the plasmid pHYBP109 which was used as the reporter plasmid and plasmid pTB1 or pTB2, which contains the *dsz* operon.

**Fig. 8:**
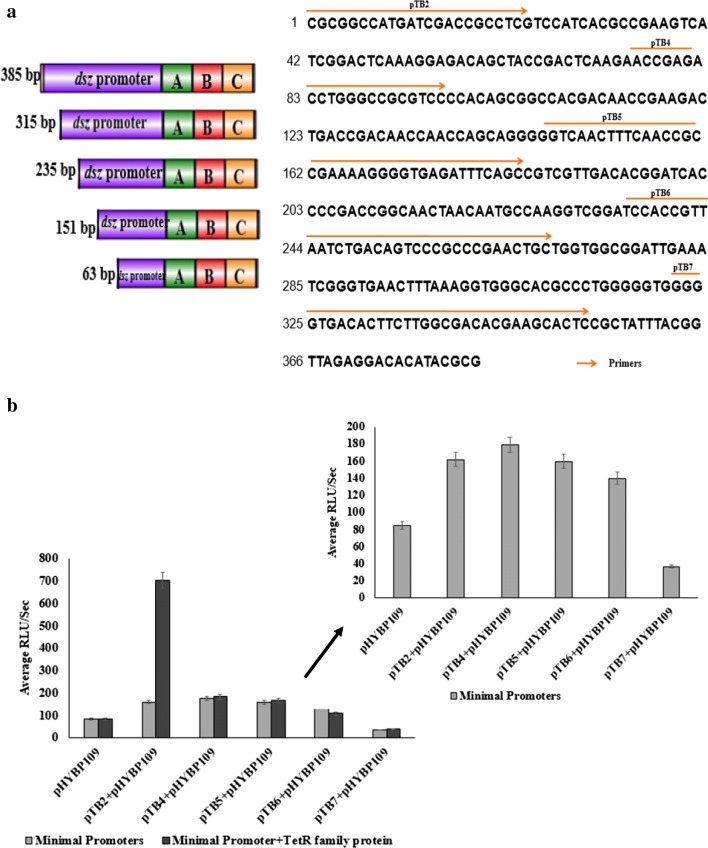
**a** Representative figure of the different deletion derivatives of the 385 bp *dsz* promoter. **b** Graph showing luciferase assay in recombinant *E. coli* BL21(DE3) pLysS harbouring full length promoter (pTB2) or deletions of promoter (pTB4–7) along with plasmid pHYBP109 (light grey bar) and in the presence of the TetR family transcription regulator (dark grey bars). Plasmid pTB2 harbour full length 385 bp promoter. Plasmid pTB4, 5, 6 and 7 contain 315 bp, 235 bp, 151 bp and 63 bp promoter fragments. Determination of minimal promoter activity using luciferase assay is represented as inset of the graph

Deletions of the promoter-regulatory fragment were made (Fig. 8a) in order to localize the minimal promoter and in an attempt to delete the regulatory regions while maintaining promoter activity. *E. coli* cells lacking any plasmid was used as negative control and it gave luciferase activity of 34 RLU/s. While *E. coli* cells harboring both pHYBP109 & pTB2 (full length promoter) was used as positive control and gave luciferase activity of around 200 RLU/s and *E. coli* cells harboring pHYBP109 alone gave luciferase activity of 89 RLU/s when cells were grown in the presence of DBT. With the increasing size of the deletions, there appeared to be complete loss of activity with the 63 bp deletion (Fig. 8b). The results suggest that the promoter must be located in the 151 bp region, as deletion beyond this eliminated promoter activity. To determine the region required for activation of the *dsz* operon in the presence of TetR family protein, these deletion constructs were used and a three-plasmid system was constructed. The TetR family gene was supplied in trans via plasmid pPM7. The three-plasmid system consisted of pHYBP109 (pBR322 ori), pTB2 (pSC101 ori) and pPM7 (p15A ori). It was found that the luciferase activities were about 200 RLU/s in control and 703 RLU/S in cells expressing TetR family protein without induction. The activities increased by approximately 15-fold in induced cells and 3.5-fold in uninduced cells. However, when pTB4 (315 bp), pTB5 (239 bp), pTB6 (151 bp), pTB7 (63 bp) were used along with TetR family protein expression plasmid, no such activation was observed in the absence of IPTG (Fig. 8b). The results suggest that the region between − 385 bp and − 315 bp contains a site for activation of the desulfurization operon.

## Discussion

For the isolation of promoter binding proteins, a number of methods have been reported. We used pull-down assay for the isolation of proteins. In the pull down assay the biotinylated *dsz* promoter is attached to the streptavidin resins packed in a column. Crude protein extract is passed through the column so that the proteins are bound to the *dsz* promoter. After washing, the bound proteins are eluted and run on a 12% SDS PAGE gel. The obtained bands were further processed and identified by MALDI-ToF. Pull down assay has been previously reported for the isolation of DNA binding proteins (Stefanovic et al. 2003). The proteins were identified by MALDI-ToF. The proteins isolated were compared with those identified by an in vivo based method (Murarka and Srivastava 2018).

It is known that the proteins interacting with the promoter DNA may have a direct or indirect role in increasing or decreasing the transcription of genes downstream to the promoter (Ptashne and Gann 1997; Ren et al. 2000). The genes encoding for the putative transcription factors which were obtained by both the methods were cloned and supplied in trans to a plasmid containing *dsz* operon. This included the TetR family protein, LuxR, DNA binding response regulator, XRE family and Fis family proteins.

The TetR family of transcriptional regulator is one of the largest and most studied groups of one component transcriptional regulators. The name is derived from the function of the first member of family, tetracycline resistance (Ramos et al. 2005b). All members of this family contain a DNA binding motif and are known to have an α-helical structure. These family of proteins may act as repressor, activator or both. LuxR regulators are another large group of transcriptional regulators. Study of a LuxR family regulator from *Vibrio harveyi* revealed that the protein can serve as both activator and repressor. Moreover, the binding site of the regulator was found to be different when serving as an activator or as a repressor. The regulator has a capability of binding to different sites in a promoter to differentially regulate gene expression (Van Kessel et al. 2013). The XRE family of transcription regulators is also reported in regulating the inducible expression by aromatic compounds and phenolic antioxidants (Ibarra et al. 2013). XRE family is similar to Cro proteins and cI repressor of bacteriophage lambda. The DNA binding response regulators are mostly part of two component system where a receiver domain senses the change in the environment and passes the information to the respective transcription regulator to activate/repress a particular pathway (Gao et al. 2007). The Fis family of proteins also known as factor of inversion was first known for their involvement in site specific recombination. However, later it was reported that this family of proteins are transcriptional regulators involved in regulation of many genes (Flåtten and Skarstad 2013). These genes are mostly associated with growth and pathways for utilization of alternate carbon and nitrogen sources (Lv et al. 2018).

Our results reveal that a protein belonging to TetR family when supplied in trans resulted in higher desulfurization activity. None of the other transcription regulators resulted in activation of the operon, instead a decrease in activity was observed. This led us to hypothesize that the TetR family protein is serving as an activator. TetR family of proteins are usually known to repress the transcription of genes (Agari et al. 2012), however there are some reports where it has been shown to serve as an activator (Ramos et al. 2005a) or perform a dual role of both activator and repressor (Cuthbertson and Nodwell 2013). LuxR has been shown to function as an activator for *lux* operon and other quorum sensing genes (Pompeani et al. 2008). DhaS activates *dha* operon (Christen et al. 2006). Another TetR family transcriptional activator DnrO has been shown to activate dnrN (Otten et al. 1995). AtrA of *Streptomyces griseus* (Hirano et al. 2008), PsrA of *Pseudomonas syringae* (Chatterjee et al. 2007), CcrR of *Methylobacterium extorquens* AM1 (Hu and Lidstrom 2012), SczA of *Streptococcus pneumonia* (Martin et al. 2017) are few other TetR family transcriptional regulators which serve as activators.

RutR exerts both positive and negative effects on promoter activity (Nguyen Le Minh et al. 2010). In general, the different members of the TetR family proteins are involved in the regulation of various genes of catabolic pathways, co-factor metabolism, lipid metabolism, amino acid metabolism, nitrogen metabolism, carbon metabolism, cell signaling, osmotic stress and many more.

Since transcription regulators are known to be required in small amounts for regulation (Balleza et al. 2009), sub optimal inducer concentrations were used in the study. We provide evidence that the TetR family transcription regulator serves as an activator of the desulfurization operon not only in heterologous host *E. coli* but also in *Gordonia* sp. IITR100 and *R. erythropolis* IGTS8. TetR family is the third most abundant family of regulators reported in bacteria (Yu et al. 2010). In *Gordonia*, there are 102 TetR family regulators (Jaishankar et al. 2017). Thus, the in vitro pull-down assay and in vivo assays were helpful in the identification of the TetR family transcription regulator that activates the desulfurization operon.

Minimal promoter was identified. Our results on identification of minimal promoter were in agreement with that of Shavandi et al. (2010). When the plasmid containing different promoter deletions was transformed with TetR family protein expression plasmid, enhanced luciferase activities were observed only when the full-length promoter was used suggesting that the site for activation lies between the − 385 and − 315 bp region.

Such a binding to the upstream region of promoter has been observed in cases of other TetR family protein activated promoters also. For example, in case of CcrR, a TetR family regulator, activation region was found in the upstream region of the promoter between positions − 334 to − 321 in *Methylobacterium extorquens* AM1 (Hu and Lidstrom 2012). Thus, such long-distance activation by TetR family regulators is not uncommon. CcrR is a TetR family protein that activates the expression of crotonyl CoA reductase/carboxylase. Crotonyl CoA reductase/carboxylase is an enzyme of the ethylmalonyl CoA pathway.

Here, we provide experimental evidence that the protein regulates the expression of desulfurization genes. It is based on the following observations: (1) TetR family protein activates the operon at sub optimal inducer concentrations (demonstrated by Gibbs assay, luciferase assay, RT-PCR and SDS-PAGE); (2) TetR family protein was detected by pull down assay when sodium sulfate extract was used suggesting that it binds to the *dsz* promoter when operon is repressed (in sodium sulfate); (3) TetR family protein was not detected in pull down assay when DBT extract was used. At present, it is not clear whether TetR family protein serves as an activator alone or it functions together with other proteins.

This is the first report on the identification of proteins that binds and activates the *dsz* promoter. A protein belonging to TetR transcription regulator family was isolated which was found to regulate the operon. It serves as an activator of operon at suboptimal inducer concentrations as observed in *Gordonia* sp. IITR100 and *R. erythropolis* IGTS8 resulting in improved biodesulfurization.

## Additional file

**Additional file 1: Figure S1.** 12% SDS-PAGE gel showing overexpression of TetR family protein. A) Expression in different expression strains. Lane1: marker, lane2: uninduced sample (BL21(DE3)), lane3: induced sample (BL21(DE3)), lane4: uninduced sample (Codon Plus), lane5: induced sample (Codon Plus), lane6: uninduced sample (BL21(DE3) pLysS), lane7: induced sample (BL21(DE3) pLysS). B) Expression of protein in BL21 DE3 pLysS at different time intervals. Lane1: marker, lane2: uninduced sample, lane3: 3 h induction sample (pellet), lane4: 3 h induction sample (supernatant), lane5: 5 h induction sample (pellet), lane6: 5 h induction sample (supernatant), lane7: 7 h induction sample (pellet), lane8: 7 h induction sample (supernatant), lane9: overnight induction sample (pellet), lane10: overnight induction sample (supernatant). **Figure S2.** Peptide fragments identified by MALDI and its similarity with the TetR family transcription regulator in *Gordonia*. **Figure S3.** Biodesulfurization activity in *E. coli* when TetR family protein is supplied in trans via plasmid demonstrated by Gibbs assay. Graph showing production of 2 HBP in cells containing pTB2 (*dsz* operon) and pTB2 + TetR induced with IPTG. The experiment was performed in triplicates. **Figure S4.** A 12% SDS-PAGE gel showing the expression of the TetR family protein when induced with different inducer concentrations. Lane1: marker, lane2: *E. coli* cells with uninduced TetR family protein, lane3-6: *E. coli* cells with induced TetR family protein (0.2 mM, 0.5 mM, 1 mM and 2 mM IPTG concentration).

## Data Availability

All data analyzed throughout this study is shown in the article.

## References

[CR1] Agari Y, Sakamoto K, Kuramitsu S, Shinkai A (2012). Transcriptional repression mediated by a TetR family protein, PfmR, from *Thermus thermophilus* HB8. J Bacteriol.

[CR2] Balleza E, López-Bojorquez LN, Martínez-Antonio A, Resendis-Antonio O, Lozada-Chávez I, Balderas-Martínez YI, Encarnación S, Collado-Vides J (2009). Regulation by transcription factors in bacteria: beyond description. FEMS Microbiol Rev.

[CR3] Chatterjee A, Cui Y, Hasegawa H, Chatterjee AK (2007). PsrA, the Pseudomonas sigma regulator, controls regulators of epiphytic fitness, quorum-sensing signals, and plant interactions in *Pseudomonas syringae* pv. tomato strain DC3000. Appl Environ Microbiol.

[CR4] Christen S, Srinivas A, Bähler P, Zeller A, Pridmore D, Bieniossek C, Baumann U, Erni B (2006). Regulation of the dha operon of *Lactococcus lactis* a deviation from the rule followed by the tetr family of transcription regulators. J Biol Chem.

[CR5] Cuthbertson L, Nodwell JR (2013). The TetR family of regulators. Microbiol Mol Biol Rev.

[CR6] Denis-Larose C, Labbe D, Bergeron H, Jones AM, Greer CW, Al-Hawari J, Grossman MJ, Sankey BM, Lau P (1997). Conservation of plasmid-encoded dibenzothiophene desulfurization genes in several rhodococci. Appl Environ Microbiol.

[CR7] Flåtten I, Skarstad K (2013). The Fis protein has a stimulating role in initiation of replication in *Escherichia coli* in vivo. PLoS ONE.

[CR8] Gao R, Mack TR, Stock AM (2007). Bacterial response regulators: versatile regulatory strategies from common domains. Trends Biochem Sci.

[CR9] Hashimoto Y, Nishiyama M, Yu F, Watanabe I, Horinouchi S, Beppu T (1992). Development of a host-vector system in a *Rhodococcus* strain and its use for expression of the cloned nitrile hydratase gene cluster. Microbiology.

[CR10] Hirano S, Tanaka K, Ohnishi Y, Horinouchi S (2008). Conditionally positive effect of the TetR-family transcriptional regulator AtrA on streptomycin production by *Streptomyces griseus*. Microbiology.

[CR11] Hu B, Lidstrom M (2012). CcrR, a TetR family transcriptional regulator, activates the transcription of a gene of the ethylmalonyl coenzyme A pathway in *Methylobacterium extorquens* AM1. J Bacteriol.

[CR12] Ibarra JA, Pérez-Rueda E, Carroll RK, Shaw LN (2013). Global analysis of transcriptional regulators in *Staphylococcus aureus*. BMC genomics.

[CR13] Jaishankar J, Singh P, Srivastava P (2017). Draft genome sequence of a biodesulfurizing bacterium, *Gordonia* sp. strain IITR100. Genome Announc.

[CR14] Jaspers MC, Suske WA, Schmid A, Goslings DA, Kohler H-PE, van der Meer JR (2000). HbpR, a new member of the XylR/DmpR subclass within the NtrC family of bacterial transcriptional activators, regulates expression of 2-hydroxybiphenyl metabolism in *Pseudomonas azelaica* HBP1. J Bacteriol.

[CR15] Konishi J, Ishii Y, Onaka T, Okumura K, Suzuki M (1997). Thermophilic carbon-sulfur-bond-targeted biodesulfurization. Appl Environ Microbiol.

[CR16] Laemmli U (1970). SDS-page Laemmli method. Nature.

[CR17] Li MZ, Squires CH, Monticello DJ, Childs JD (1996). Genetic analysis of the dsz promoter and associated regulatory regions of *Rhodococcus erythropolis* IGTS8. J Bacteriol.

[CR18] Li GQ, Li SS, Zhang ML, Wang J, Zhu L, Liang FL, Liu RL, Ma T (2008). Genetic rearrangement strategy for optimizing the dibenzothiophene biodesulfurization pathway in Rhodococcus erythropolis. Appl Environ Microbiol.

[CR19] Lv M, Chen Y, Liao L, Liang Z, Shi Z, Tang Y, Ye S, Zhou J, Zhang L (2018). Fis is a global regulator critical for modulation of virulence factor production and pathogenicity of *Dickeya zeae*. Scientific Rep.

[CR20] Martin JE, Edmonds KA, Bruce KE, Campanello GC, Eijkelkamp BA, Brazel EB, McDevitt CA, Winkler ME, Giedroc DP (2017). The zinc efflux activator SczA protects *Streptococcus pneumoniae* serotype 2 D39 from intracellular zinc toxicity. Mol Microbiol.

[CR21] Mohebali G, Ball AS (2016). Biodesulfurization of diesel fuels–past, present and future perspectives. Int Biodeterior Biodegrad.

[CR22] Murarka P, Srivastava P (2018). An improved method for the isolation and identification of unknown proteins that bind to known DNA sequences by affinity capture and mass spectrometry. PLoS ONE.

[CR23] Nguyen Le Minh P, Bervoets I, Maes D, Charlier D (2010). The protein–DNA contacts in RutR carAB operator complexes. Nucleic Acids Res.

[CR24] Oldfield C, Wood NT, Gilbert SC, Murray FD, Faure FR (1998). Desulphurisation of benzothiophene and dibenzothiophene by actinomycete organisms belonging to the genus *Rhodococcus*, and related taxa. Antonie Van Leeuwenhoek.

[CR25] Otten SL, Ferguson J, Hutchinson CR (1995). Regulation of daunorubicin production in *Streptomyces peucetius* by the dnrR2 locus. J Bacteriol.

[CR26] Pompeani AJ, Irgon JJ, Berger MF, Bulyk ML, Wingreen NS, Bassler BL (2008). The Vibrio harveyi master quorum-sensing regulator, LuxR, a TetR-type protein is both an activator and a repressor: DNA recognition and binding specificity at target promoters. Mol Microbiol.

[CR27] Ptashne M, Gann A (1997). Transcriptional activation by recruitment. Nature.

[CR28] Ramos JL, Martínez-Bueno M, Molina-Henares AJ, Terán W, Watanabe K, Zhang X, Gallegos MT, Brennan R, Tobes R (2005). The TetR family of transcriptional repressors. Microbiol Mol Biol Rev.

[CR29] Ramos JL, Martínez-Bueno M, Molina-Henares AJ, Terán W, Watanabe K, Zhang X, Gallegos MT, Brennan R, Tobes RJMMBR (2005). The TetR family of transcriptional repressors. Microbial Mol Biol Rev.

[CR30] Ren B, Robert F, Wyrick JJ, Aparicio O, Jennings EG, Simon I, Zeitlinger J, Schreiber J, Hannett N, Kanin E (2000). Genome-wide location and function of DNA binding proteins. Science.

[CR31] Sambrook J, Russell D (2001). Molecular cloning: a laboratory manual.

[CR32] Shavandi M, Sadeghizadeh M, Khajeh K, Mohebali G, Zomorodipour A (2010). Genomic structure and promoter analysis of the dsz operon for dibenzothiophene biodesulfurization from *Gordonia alkanivorans* RIPI90A. Appl Microbiol Biotechnol.

[CR33] Shevchenko A, Wilm M, Vorm O, Mann M (1996). Mass spectrometric sequencing of proteins from silver-stained polyacrylamide gels. Anal Chem.

[CR34] Singh P, Srivastava P (2013). An improved protocol for electroporation in members of the genus *Gordonia*. J Microbiol Methods.

[CR35] Singh P, Chachan S, Singhi D, Srivastava PJG (2016). Isolation and molecular characterization of a stationary phase promoter useful for gene expression in Gordonia. Gene.

[CR36] Srivastava P, Fekete RA, Chattoraj DK (2006). Segregation of the replication terminus of the two *Vibrio cholerae* chromosomes. J Bacteriol.

[CR37] Stefanovic D, Stanojcic S, Vindigni A, Ochem A, Falaschi A (2003). In vitro protein-DNA interactions at the human lamin B2 replication origin. J Biol Chem.

[CR38] Thakur CS, Brown ME, Sama JN, Jackson ME, Dayie TK (2010). Growth of wildtype and mutant *E. coli* strains in minimal media for optimal production of nucleic acids for preparing labeled nucleotides. Appl Microbiol Biotechnol.

[CR39] Van Kessel JC, Ulrich LE, Zhulin IB, Bassler BL (2013). Analysis of activator and repressor functions reveals the requirements for transcriptional control by LuxR, the master regulator of quorum sensing in *Vibrio harveyi*. MBio.

[CR40] Yamamoto K, Nishimura M, D-i Kato, Takeo M, Negoro S (2011). Identification and characterization of another 4-nitrophenol degradation gene cluster, nps, in *Rhodococcus* sp. strain PN1. J Biosci Bioeng.

[CR41] Yu Z, Reichheld SE, Savchenko A, Parkinson J, Davidson AR (2010). A comprehensive analysis of structural and sequence conservation in the TetR family transcriptional regulators. J Mol Biol.

