# Supplementary material for: Isolation and identification of a TetR family protein that regulates the biodesulfurization operon
Source: AMB Express. 2019 May 24;9:71. doi: 10.1186/s13568-019-0801-x (PMC6534649; doi:10.1186/s13568-019-0801-x)
Supplement: Supplementary file 1 — Additional file 1: Figure S1. 12% SDS-PAGE gel showing overexpression of TetR family protein. A) Expression in different expression strains. Lane1: marker, lane2: uninduced sample (BL21(DE3)), lane3: induced sample (BL21(DE3)), lane4: uninduced sample (Codon Plus), lane5: induced sample (Codon Plus), lane6: uninduced sample (BL21(DE3) pLysS), lane7: induced sample (BL21(DE3) pLysS). B) Expression of protein in BL21 DE3 pLysS at different time intervals. Lane1: marker, lane2: uninduced sample, lane3: 3 h induction sample (pellet), lane4: 3 h induction sample (supernatant), lane5: 5 h induction sample (pellet), lane6: 5 h induction sample (supernatant), lane7: 7 h induction sample (pellet), lane8: 7 h induction sample (supernatant), lane9: overnight induction sample (pellet), lane10: overnight induction sample (supernatant). Figure S2. Peptide fragments identified by MALDI and its similarity with the TetR family transcription regulator in Gordonia. Figure S3. Biodesulfurization activity in E. coli when TetR family protein is supplied in trans via plasmid demonstrated by Gibbs assay. Graph showing production of 2 HBP in cells containing pTB2 (dsz operon) and pTB2 + TetR induced with IPTG. The experiment was performed in triplicates. Figure S4. A 12% SDS-PAGE gel showing the expression of the TetR family protein when induced with different inducer concentrations. Lane1: marker, lane2: E. coli cells with uninduced TetR family protein, lane3-6: E. coli cells with induced TetR family protein (0.2 mM, 0.5 mM, 1 mM and 2 mM IPTG concentration). [file 13568_2019_801_MOESM1_ESM.pdf]

Additional file 1

Journal: AMB Express

Isolation and identification of a TetR family protein that regulates the biodesulfurization operon

Pooja Murarka, Tanaya Bagga, Pooja Singh, Sabita Rangra and Preeti Srivastava*

Department of Biochemical engineering & Biotechnology, Indian Institute of Technology Delhi, New Delhi, India

*** Corresponding author**

preeti@dbeb.iitd.ac.in, preetisrivastava@hotmail.com

Additional file 1: Supplementary Figures

Figure S1 12% SDS-PAGE gel showing overexpression of TetR family protein. A) Expression in different expression strains. Lane1: marker, lane2: uninduced sample (BL21(DE3)), lane3: induced sample (BL21(DE3)), lane4: uninduced sample (Codon Plus), lane5: induced sample (Codon Plus), lane6: uninduced sample (BL21(DE3) pLysS), lane7: induced sample (BL21(DE3) pLysS). B) Expression of protein in BL21 DE3 pLysS at different time intervals. Lane1: marker, lane2: uninduced sample, lane3: 3hr induction sample (pellet), lane4: 3hr induction sample (supernatant), lane5: 5hr induction sample (pellet), lane6: 5hr induction sample (supernatant), lane7: 7hr induction sample (pellet), lane8: 7hr induction sample (supernatant), lane9: overnight induction sample (pellet), lane10: overnight induction sample (supernatant).

Figure S2 Peptide fragments identified by MALDI and its similarity with the TetR family transcription regulator in *Gordonia*

Figure S3 Biodesulfurization activity in *E. coli* when TetR family protein is supplied in trans via plasmid demonstrated by Gibbs assay. Graph showing production of 2 HBP in cells containing pTB2 (*dsz* operon) and pTB2+ TetR induced with IPTG. The experiment was performed in triplicates.

Figure S4 A 12% SDS-PAGE gel showing the expression of the TetR family protein when induced with different inducer concentrations. Lane1: marker, lane2: *E. coli* cells with uninduced TetR family protein, lane3-6: *E. coli* cells with induced TetR family protein (0.2 mM, 0.5 mM, 1 mM and 2 mM IPTG concentration)

A

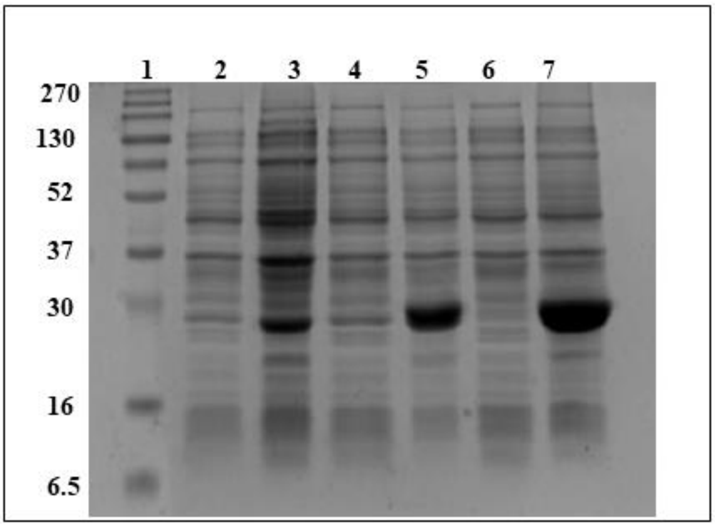

1 2 3 4 5 6 7
270
130
52
37
30
16
6.5

B

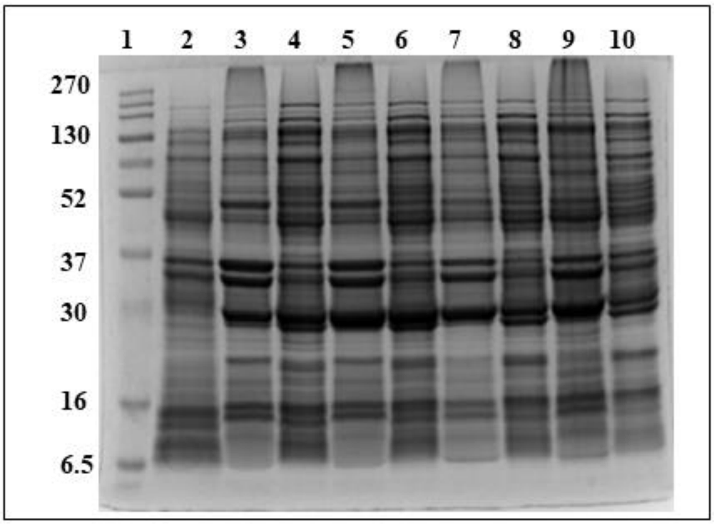

1 2 3 4 5 6 7 8 9 10
270
130
52
37
30
16
6.5

Figure S1

Probability Based Mowse Score

Protein score is -10*Log(P), where P is the probability that the observed match is a random event.
Protein scores greater than 72 are significant (p<0.05).
Protein scores are derived from ions scores as a non-probabilistic basis for ranking protein hits.

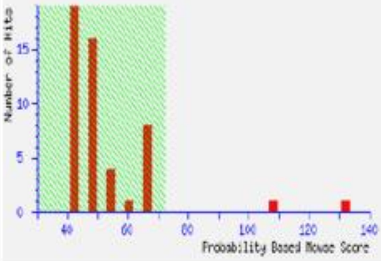

	Accession	Mass	Score	Description
1.	gi|326384056	22645	132	regulatory protein TetR [Gordonia neofelifaecis

Protein sequence coverage: 21%

Matched peptides shown in ***bold red***.

1 MTPPPADDTS GKSGRRTELL DIAATLFAER **GLRATTVR**DI ADAAGILSGS
51 LYHHFDSKES MVDEILRGFL DDLFGKYREI VASGLDSRAT LEALVTTSYE
101 AIDASHSAVA IYQDEVKHLV ANERFTYLSE LNTEFR**ELWM** **GVLEAGVK**DG
151 SFRSDIDVEL AFRFLR**DTAW** **VAVR**WYRPGG SVTVDTVAK**Q** **YLSIVLDGLA**
201 **SPHN**

>gi|498805763|ref|WP_010840674.1| TetR family transcriptional regulator [*Gordonia* sp. IITR100]
MSPRGQTRIDANGRTAASTADAPTRSSRRDEL
LATAGRMFAEQGLRSTTVRDIADAAGILSGSL
YHHFDSKESMVDEILRGFLDDLFARYRQIAAS
SKSATETLRGLVIASFESIDAERNAVAIYQDEA
KRLSGQERFAYISELNVEFRQLWQSVLQRGVE
NGEFRADLDVELVYRFMRDTVWVAVRWYRP
GGSMTVDSIADQYLSVVLD GILPR

- BLAST shows 68% similarity with TetR family transcriptional regulator in *Gordonia*.

Figure S2

A

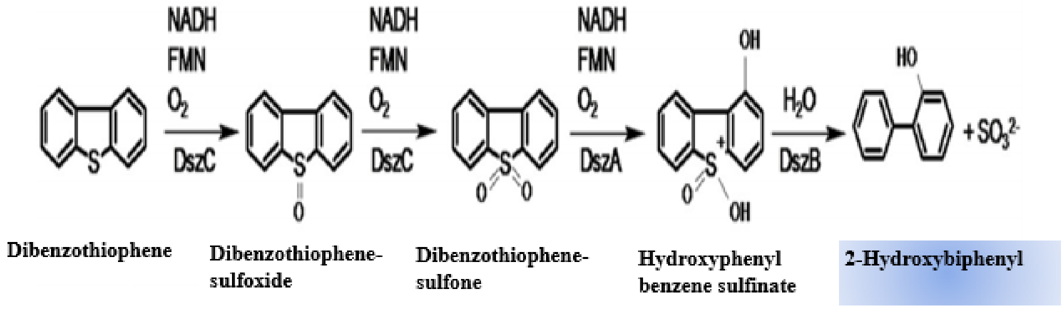
NADH
FMN
O_2
DszC
NADH
FMN
O_2
DszC
NADH
FMN
O_2
DszA
H_2O
DszB
$+SO_3^{2-}$
Dibenzothiophene
Dibenzothiophene-sulfoxide
Dibenzothiophene-sulfone
Hydroxyphenyl benzene sulfinate
2-Hydroxybiphenyl

B

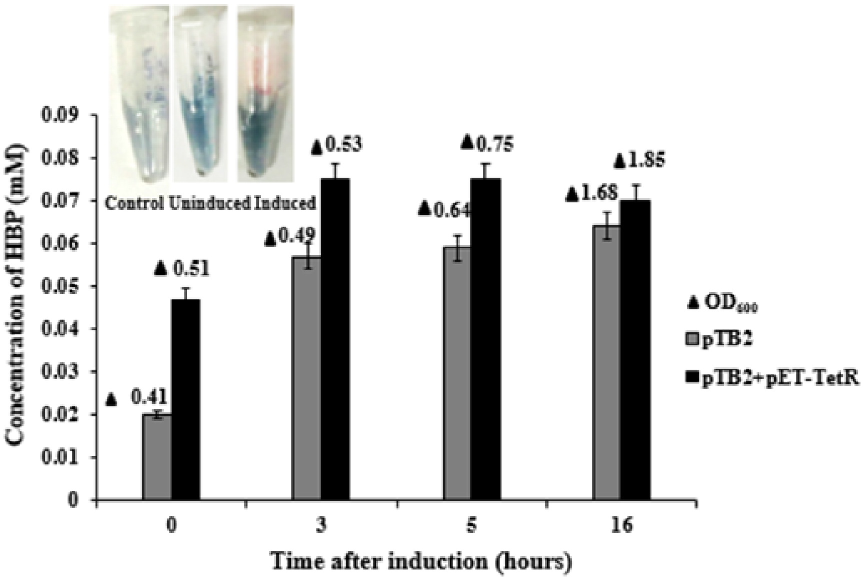
Control Uninduced Induced
Concentration of HBP (mM)
0.09
0.08
0.07
0.06
0.05
0.04
0.03
0.02
0.01
0
0.41
0.51
0.49
0.53
0.64
0.75
1.68
1.85
0
3
5
16
Time after induction (hours)
OD_{600}
pTB2
pTB2+pET-TetR

Figure S3

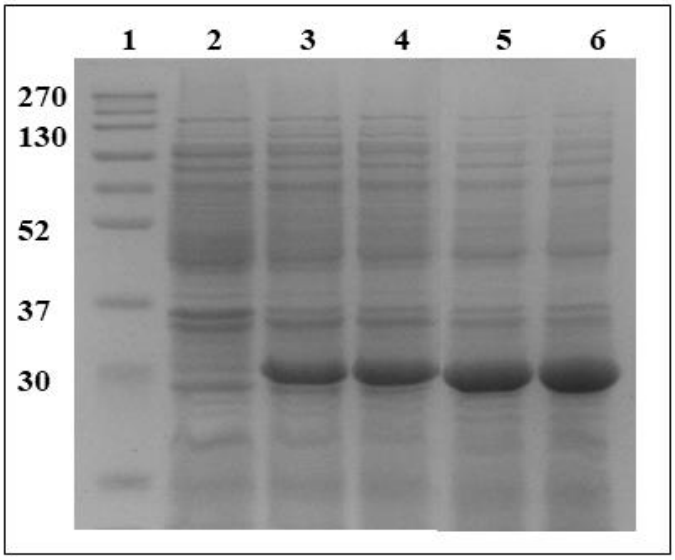

Figure S4
